# Subcortical and default mode network connectivity is impaired in myalgic encephalomyelitis/chronic fatigue syndrome

**DOI:** 10.3389/fnins.2023.1318094

**Published:** 2024-01-29

**Authors:** Maira Inderyas, Kiran Thapaliya, Sonya Marshall-Gradisnik, Markus Barth, Leighton Barnden

**Affiliations:** ^1^National Centre for Neuroimmunology and Emerging Diseases, Menzies Health Institute Queensland, Griffith University, Southport, QLD, Australia; ^2^School of Information Technology and Electrical Engineering, The University of Queensland, Brisbane, QLD, Australia

**Keywords:** fMRI, myalgic encephalomyelitis/chronic fatigue syndrome, ME/CFS, functional connectivity, 7 Tesla MRI, CONN, impaired memory, duration of illness

## Abstract

Myalgic encephalomyelitis/chronic fatigue syndrome (ME/CFS) is a complex chronic condition with core symptoms of fatigue and cognitive dysfunction, suggesting a key role for the central nervous system in the pathophysiology of this disease. Several studies have reported altered functional connectivity (FC) related to motor and cognitive deficits in ME/CFS patients. In this study, we compared functional connectivity differences between 31 ME/CFS and 15 healthy controls (HCs) using 7 Tesla MRI. Functional scans were acquired during a cognitive Stroop color-word task, and blood oxygen level-dependent (BOLD) time series were computed for 27 regions of interest (ROIs) in the cerebellum, brainstem, and salience and default mode networks. A region-based comparison detected reduced FC between the pontine nucleus and cerebellum vermis IX (*p = 0.027*) for ME/CFS patients compared to HCs. Our ROI-to-voxel analysis found significant impairment of FC within the ponto-cerebellar regions in ME/CFS. Correlation analyses of connectivity with clinical scores in ME/CFS patients detected associations between FC and ‘duration of illness’ and ‘memory scores’ in salience network hubs and cerebellum vermis and between FC and ‘respiratory rate’ within the medulla and the default mode network FC. This novel investigation is the first to report the extensive involvement of aberrant ponto-cerebellar connections consistent with ME/CFS symptomatology. This highlights the involvement of the brainstem and the cerebellum in the pathomechanism of ME/CFS.

## 1 Introduction

Myalgic encephalomyelitis, also known as chronic fatigue syndrome (ME/CFS), is a chronic debilitating disease with key symptoms of cognitive dysfunction, profound and pervasive fatigue that fails to improve with rest (Cortes Rivera et al., 2019), unrefreshing sleep, and post-exertional malaise (PEM) that lasts for more than 6 months (Jason et al., 2014). ME/CFS affects approximately 17–24 million people worldwide (Lim and Son, 2020). The etiology and the pathomechanism of ME/CFS remain largely elusive, with no universal diagnostic tests available (Jason et al., 2014; Cortes Rivera et al., 2019; Lim and Son, 2020). Diagnosis is made according to the Canadian Consensus Criteria (CCC) (Carruthers et al., 2003) and the International Consensus Criteria (ICC) (Carruthers et al., 2011).

Magnetic resonance imaging (MRI) studies have been used to study structural and functional changes in ME/CFS patients. Anatomical anomalies have been reported in cortical and subcortical regions (Buchwald et al., 1992; Okada et al., 2004; de Lange et al., 2005), with a significant reduction in cortical volume and thickness and an increase in hippocampal and brainstem volumes in ME/CFS patients when compared with healthy controls (HCs) (Thapaliya et al., 2022). Moreover, Thapaliya et al. (2020) reported significantly higher signal intensities in the white matter and basal ganglia regions using the ratio of T1 weighted-T2 weighted images in ME/CFS patients. One longitudinal study reported progressive regional white matter (WM) volume reduction in ME/CFS patients (Shan et al., 2016), while a diffusion tensor imaging (DTI) study observed changes in the descending cortico-cerebellar tract in the midbrain and pons (Thapaliya et al., 2021).

Functional MRI (fMRI) studies have reported altered blood oxygen level-dependent (BOLD) responses in ME/CFS patients (Tanaka et al., 2006; Shan et al., 2018a). Myelin-sensitive studies have demonstrated abnormal BOLD associations with clinical scores in ME/CFS patients (Barnden et al., 2011, 2015, 2016) and enhanced myelin relative to HCs in sensorimotor white matter (Barnden et al., 2018). There is evidence that individual differences in functional connectivity (FC) are associated with cognitive and behavioral changes (Tanaka et al., 2006). Altered brain functions have been observed between various regions of the brain in ME/CFS, including different cortical regions, resulting in motor planning dysfunction (de Lange et al., 2004), altered FC in the primary intrinsic networks (Boissoneault et al., 2016; Shan et al., 2018b), and basal ganglia dysfunction (Miller et al., 2014). Another study observed impaired FC within the brainstem between the medulla and cuneiform nucleus (Barnden et al., 2019). Abnormal FC in the default mode network (DMN), salience network (SA), and affective networks was reported (Tanaka et al., 2006; Kim et al., 2015; Gay et al., 2016; Shan et al., 2018b; Su et al., 2023) for both the resting state and during cognitive tasks in ME/CFS patients. Lower FC in the intrinsic networks in ME/CFS patients (Boissoneault et al., 2016; Zinn et al., 2016; Manca et al., 2021; Rayhan and Baraniuk, 2021; Barnden et al., 2023; Su et al., 2023) could lead to deficits in executive function, such as memory, information processing speed, learning ability, and overall neurocognition (Menon, 2011). Hence, individual differences in connectivity patterns are important for cognitive and behavioral functions, with the brainstem and the cerebellum being particularly involved in cognitive impairment, motor dysfunction, and the sleep–wake cycle (Song and Zhu, 2021).

Therefore, we hypothesised that, in ME/CFS patients, FC is altered in two intrinsic networks, the salience network (SA) and default mode network (DMN), and the cerebellar vermis and brainstem regions. We used an ultra-high field 7 Tesla MRI, which provides high spatial resolution and improved signal-to-noise BOLD signals (van der Zwaag et al., 2012; Colizoli et al., 2022). This study aimed to use ultra-high field 7 T MRI to (a) study ME/CFS differences in FC among SA, DMN, cerebellum, and pontine nuclei regions compared with HCs and (b) explore the relationship between clinical measures and FC in ME/CFS patients.

## 2 Materials and methods

### 2.1 Participant recruitment

This study was approved by the Griffith University Human Research Ethics Committee (2022/666), and written informed consent was obtained from all participants. This cross-sectional study was conducted at the National Centre of Neuroimmunology and Emerging Diseases (NCNED) on the Gold Coast, Queensland, Australia. We recruited 31 ME/CFS patients (see Table 1), fulfilling the CCC and/or ICC definitions of diagnosis (Carruthers et al., 2003, 2011). All ME/CFS patients were referred by a medical practitioner and assessed through NCNED research questionnaires for their symptom severity scores. A total of 15 HCs, aged between 18 and 65 years (see Table 1), were recruited for the study. All HCs had expressed their interest in volunteering via email, telephone, social media, and/or through friends/family referrals. Healthy individuals with no reported chronic health condition or evidence of underlying illness or conditions such as mental illness, malignancies, autoimmune, neurological, and cardiovascular diseases, and who were pregnant and/or breastfeeding were contacted. All participants were asked to complete an online registry questionnaire to be screened by NCNED researchers to evaluate whether volunteers were eligible to be included in our study.

**Table 1:** Demographic information for healthy controls and ME/CFS participants and clinical scores for 31 ME/CFS patients were used for correlation analysis to test for functional connectivity differences.

	Age	F/M	*p*-value	Duration illness	Impaired memory	Respiratory rate
HC (*n* = 15)	38.2 ± 12.7	10/5	0.24	N/A	N/A	N/A
ME/CFS (*n* = 31)	43.1 ± 10.9	24/7	0.40	12.3 ± 11.0	2.7 ± 1.5	3.8 ± −1.1

N/A denotes no scores for HC.

### 2.2 MRI acquisition

Functional MRI was acquired as in Barnden et al. (2023) on a 7 T whole-body MRI scanner (Siemens Healthcare, Erlangen, Germany) with a 32-channel coil (Nova MRI Wilmington, NC, United States). The data were acquired sagittally using a multiband echo-planar imaging (EPI) pulse sequence developed at the University of Minnesota (Auerbach et al., 2013) at a multiband factor of 3, repetition time (TR) of 2,000 ms, echo time (TE) of 22.4 ms, a flip angle of 70^o^, an interleaved multi-slice mode, an acquisition matrix of 192×192, and a voxel size of 1.25 mm^3^. We acquired 225 volumes comprising 80 sagittal slices, while both study groups, 31 ME/CFS patients and 15 HCs, responded to a sequence of Stroop color-word tests.

T1-weighted data were also sagittally acquired on the same 7 T scanner using a Magnetization Prepared 2 Rapid Acquisition Gradient Echo Sequence (MP2RAGE) as described by Thapaliya et al. (2019) with the following parameters: repetition time (TR) = 4,300 ms; echo time (TE) = 2.45 ms; first inversion time (TI1) = 840 ms; second inversion time (TI2) = 2,370 ms; first flip angle (FA1) = 5^o^; second flip angle (FA2) = 6^o^; and resolution = 0.75 mm^3^ with a matrix size of 256x300x320.

### 2.3 Stroop task

The Stroop task was used in fMRI to investigate concentration and attention difficulties (Barnden et al., 2023). For each task, two colored words were displayed. The upper word, “Blue”, “Red”, “Yellow”, “Green”, or “XXXX”, was colored red, green, blue, or yellow on a black background. The lower word, “Blue”, “Red”, “Yellow”, or “Green”, was colored white on a black background. Participants were asked to decide whether the color of the upper word agreed with the meaning of the lower word and to press one of the two buttons on a handpiece to respond “yes” or “no”. The Stroop task fMRI was divided into four conditions, of which three were trials: neutral (upper word “XXXX”), incongruent (upper word written in a different color from its meaning), or congruent (upper word written in the color of its meaning). Due to conflict resolution in deciding the meaning of the upper word vs. the meaning of the lower word rather than color vs. meaning, i.e., the natural impulse to read the upper word before inspecting its color, the incongruent task was relatively more challenging than the congruent. The fourth condition, rest, refers to the pause between a trial response and the next trial onset, during which a stationary cross appears on the screen for a randomized period between 3 and 12 s. In total, 60 Stroop trials were randomly distributed over 7.5 min; 40% of the trials were incongruent, 30% were congruent, and 30% were neutral, and the average inter-stimulus time was 10.5 s. For each trial, the stimulus onset and response times were recorded, and the difference between them was the reaction time. Mean reaction time and accuracy were estimated as in the study by Barnden et al. (2023) for each trial for each subject group.

### 2.4 Clinical scores

Clinical symptom scores were extracted from the 36-Item Short Form Health Survey (Alonso et al., 1995) within the research registry questionnaire developed by NCNED with the Centers for Disease Control and Prevention (CDC), which was accessed online through the LimeSurvey. Validated patient-reported outcome measures were used to determine participants’ quality of life (QoL) and functional capacity. The clinical parameters “duration of illness” and self-reported “memory scores” were correlated with FC in ME/CFS patients. The severity of symptoms for impaired memory was assessed on a 5-point scale: (1) very mild; (2) mild; (3) moderate; (4) severe; and (5) very severe. Symptom severity was reported as the median severity score on the 5-point severity scale for each symptom. The frequency of each symptom experienced was also reported on a 5-point scale: (1) a little of the time; (2) some of the time; (3) good bit of the time; (4) most of the time; or (5) all the time. Finally, the duration of symptoms was assessed according to self-reported data as months and years. Autonomic “respiratory rate” (Resp) was extracted from the power spectra of a pulse oximeter and respiration strap time series recorded during the 7.5 min fMRI (peak frequency and full width of peak at half maximum) as in the study by Barnden et al. (2023) (see Table 1). All the demographic and clinical measures of individuals are provided as Supplementary material.

### 2.5 Regions of interest

We examined 27 regions of interest (ROIs) to compare FC between ME/CFS patients and HCs (see Table 2). Of these 27 ROIs, 7 (anterior insula, supramarginal gyrus, rostral prefrontal, and anterior cingulate cortices) were salience network (SA) hubs; 4 (inferior lateral parietal, medial prefrontal, and posterior cingulate cortices) were default mode network (DMN) hubs; 7 were cerebellar regions (6 vermis and culmen), and 5 were in the brainstem. We limited our analysis to only 27 ROIs to avoid excessive multiple-comparison corrections for statistical inference. All ROIs with their laterality and number of voxels are shown in Table 2.

**Table 2:** List of ROIs used to test the functional connectivity between HCs and ME/CFS patients.

ROI location	Laterality	Abbreviation
Default mode network (4)		
Inferior lateral parietal	L&R	DM.LP(L)
		DM.LP(R)
Medial prefrontal cortex	Midline	DM.MPFC
Posterior cingulate cortex	Midline	DM.PCC
Salience network (7)		
Anterior insula	L&R	SA.AI_L
		SA.AI_R
Supramarginal gyrus	L&R	SA.SMG_L
		SA.SMG_R
Rostral prefrontal cortex	L&R	SA.RPFC_L
		SA.RPFC_R
Anterior cingulate cortex	Midline	SA.ACC
Cerebellum (7)		
Declive		Vermis_VI
Tuber		Vermis_VIIb
Pyramis		Vermis_VIIIa
		Vermis_VIIIb
Uvula		Vermis_IX
Nodulus		Vermis_X
Crus II		Vermis_crus_II
Culmen of cerebellum		Culmn
Brainstem (5)		
Paramedian reticular nucleus		PMnR
Pontine reticular nucleus, oral and caudal (pontis oralis and caudalis)	L&R	PnO_PnC_L
		PnO_PnC_R
Cuneiform nucleus	L&R	CnF_L
		CnF_R
Rostral medula	L&R	Mdul_L
		Mdul_R
Pontine nuclei	L&R	PoNucl_L
		PoNucl_R

L, left, R, right.

### 2.6 MRI processing

#### 2.6.1 Pre-processing

Functional and anatomical data were pre-processed using the CONN toolbox (Whitfield-Gabrieli and Nieto-Castanon, 2012) release 22.a based on SPM 12, http://www.fil.ion.ucl.ac.uk/spm/software/spm12/, in MATLAB version R2019b (MathWorks, Natick, Massachusetts).

In brief, a default pre-processing pipeline (Nieto-Castanon, 2020) was used that included realignment with the correction of susceptibility distortion interactions, slice timing correction, outlier detection, direct segmentation, and MNI-space normalization, smoothing, and the removal of initial scans. According to Andersson et al. (2001), functional data were realigned and unwarped in SPM, co-registered to the first scan of the first session (reference image) (Nagao, 2004; Friston, 2009), and resampled for motion correction and magnetic susceptibility interactions followed by an SPM slice-timing correction (STC) procedure (Sladky et al., 2011) for temporal misalignment correction to resample BOLD time series from each slice to a common mid-acquisition time. Potential outlier scans were identified using Artifact Detection Tools (ARTs) with framewise displacement above 0.9 mm or global BOLD signal changes above 5 standard deviations (Power et al., 2014), and a reference BOLD image was computed for each subject by averaging all scans excluding outliers. Spatial normalization of functional and anatomical data into standard MNI space was performed, followed by segmentation into gray matter, white matter, and CSF tissue classes, and resampling to 1 mm isotropic voxels (Calhoun et al., 2017) using SPM’s unified segmentation and normalization algorithm (Ashburner, 2007). fMRI volumes were smoothed using spatial convolution with a Gaussian kernel of 5 mm full-width half maximum (FWHM), and the first five scans in each functional run were removed to allow the stabilization of the magnetic field.

#### 2.6.2 Denoising

CONN’s default denoising pipeline was used (Nieto-Castanon, 2020) and regressed for potential confounding effects characterized by white matter time series, CSF time series, motion parameters, and their first-order derivatives using the Component Correction (CompCor) method (Friston et al., 1995; Behzadi et al., 2007; Chai et al., 2012). Outlier scans, session and task effects, and their first-order derivatives and linear trends were also included as linear confounding effects. Bandpass frequency filtering of the BOLD time series (Hallquist et al., 2013) was between 0.008 Hz and 0.09 Hz, and the effective degrees of freedom of the BOLD signal after denoising were estimated to range from 55.8 to 63.6 (average 63) across all participants (Nieto-Castanon, 2020).

#### 2.6.3 Statistical analyses: first-level and group-level

ROI-to-ROI connectivity (RRC) matrices and seed-to-voxel (SBC) maps were estimated by characterizing the patterns of functional connectivity with 27 ROIs. Functional connectivity strength was represented by Fisher-transformed bivariate correlation coefficients from a weighted general linear model (weighted-GLM) (Nieto-Castanon, 2020) and defined separately for each pair of seed and target areas, modeling the association between their BOLD signal time series. To compensate for possible transient magnetization effects at the beginning of each run, individual scans were weighted by a step function convolved with an SPM canonical hemodynamic response function and rectified.

Group-level analyses were performed using a GLM (Nieto-Castanon, 2020). For each individual voxel, a separate GLM was estimated, with first-level connectivity measures at this voxel as dependent variables (one independent sample per subject and one measurement per task or experimental condition, if applicable) and groups or other subject-level identifiers as independent variables. Voxel-level hypotheses were evaluated using multivariate parametric statistics with random effects across subjects and sample covariance estimation across multiple measurements.

##### 2.6.3.1 Statistical inference

Inferences were performed at the level of individual clusters (groups of contiguous voxels). Cluster-level inferences were based on parametric statistics from the Gaussian Random Field theory (Worsley, 1997; Nieto-Castanon, 2020).

The results were obtained from CONN as the T statistic for connectivity difference for each ROI pair and a false discovery rate (FDR) corrected value of *p* (Benjamini and Hochberg, 1995) defined as the expected proportion of false discoveries among all ROI pairs with similar or larger effects. Each ROI pair with an FDR corrected value of *p* < 0.05 for the difference between ME and HC is reported below. The results for the seed-to-voxel tests for significant clusters were thresholded using a combination of a cluster-forming *p* < 0.001 voxel-level threshold and a familywise corrected p-FDR of <0.05 for the cluster-size threshold (Nieto-Castanon, 2020).

## 3 Results

### 3.1 ROI-to-ROI connectivity

**Figure 1:**
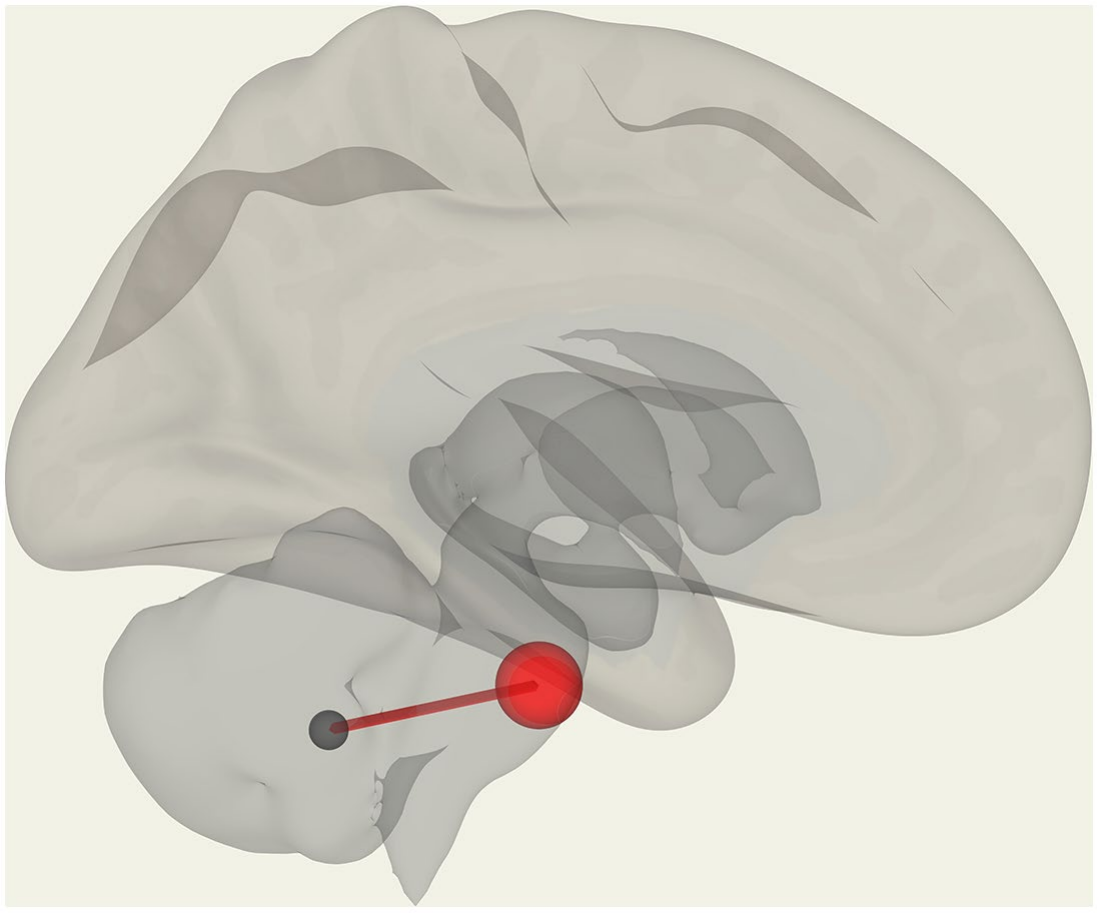
Reduced FC in ME/CFS patients compared to HCs between the pontine nucleus and cerebellar vermis IX.

We observed significantly reduced functional connectivity (*p* = 0.027) between vermis IX and pontine nuclei (PoNucl) in ME/CFS patients compared to HCs, as shown in Figure 1.

### 3.2 Seed-based connectivity

**Figure 2:**
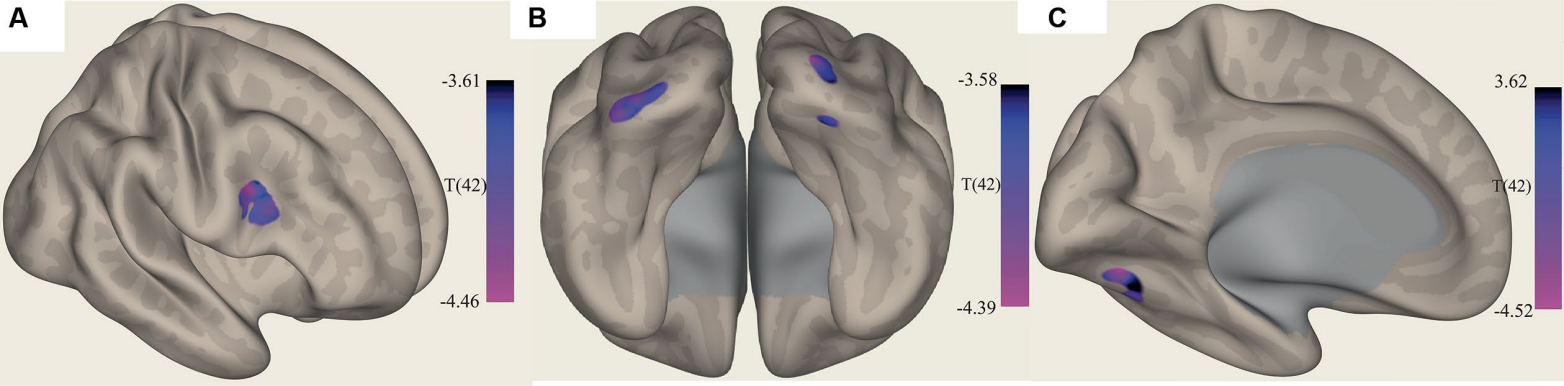
Clusters with increased FC in ME/CFS patients. **(A)** Shows the voxel cluster in the Inf. frontal gyrus with increased ME/CFS connectivity to the cerebellum vermis VI ROI. **(B)** Shows a posterio-inferior view of three voxel clusters in the right cerebellum and occipital pole (left and right with increased ME/CFS connectivity to the left cuneiform nucleus ROI). **(C)** Shows a voxel cluster in left cerebellum VI with increased ME/CFS connectivity to the culmen region; T(42) thresholded >3.54.

**Figure 3:**
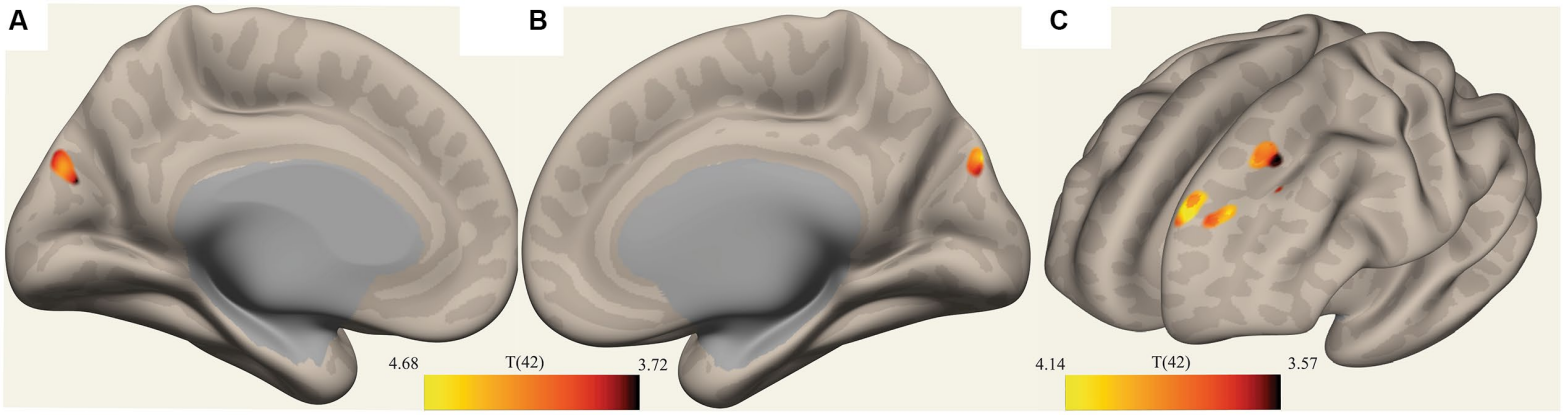
Clusters with reduced FC in ME/CFS patients. **(A,B)** Show a voxel cluster in the cuneus with decreased ME/CFS connectivity with the default mode network PCC ROI, while **(C)** Shows two voxel clusters in the left frontal pole and left suprafrontal gyrus with decreased ME/CFS connectivity with the left pontine nucleus ROI; T(42) thresholded >3.54.

The ROI-to-voxel analysis found that FC was significantly different between several ROIs and voxel clusters (see Table 3 and Figures 2, 3). Reduced FC in ME/CFS patients was seen between the vermis VI ROI and a right inferior frontal gyrus (IFG) cluster (*p* = 0.002); between the left cuneiform ROI and left/right occipital pole (OP) (left; *p* = 0.002; right; *p* = 0.023); and between the culmen ROI and left cerebellum 6 (*p* = 0.0001) (see Table 3 and Figures 2, 3). Stronger FC in ME/CFS was observed between the DMN’s posterior cingulate cortex (PCC) ROI and cuneus (*p* = 0.007) and between the PoNucl ROI and left frontal pole (*p* = 0.007) and left superior frontal gyrus (*p* = 0.046).

**Table 3:** ROI-to-voxel results.

ROI	ME/CFS	Cluster location	x y z (MNI)	*P_uncorr_* (0.001)	k
Vermis_VI	↓	IFG oper_R	+44 + 10 + 26	0.002	82
CnF_L	↓	OP _L	−40 –90 -04	0.002	73
	↓	OP_R	+26–102 + 06	0.023	50
	↓	CerebI R	+30–80 -22	0.040	45
Culmn	↓	CerebVI_L	−28 –68 − 18	0.0001	121
DMN_PCC	↑	Cuneous	+02–80 + 38	0.007	76
PoNucl_L	↑	FP _L	-18 + 54 + 38	0.0077	68
	↑	SFG_L	−22 + 32 + 54	0.0461	43

The “ME/CFS” column indicates ME/CFS FC was weaker (↓) or stronger (↑) than HCs. Cluster statistics (p-FDR) and size (k voxels) for uncorrected voxel thresholds of *P_uncorr_* < 0.001 are listed. CnF_L = left cuneiform, Culmn = Culmen, DMN_PCC = posterior cingulate cortex of the default mode network, IFG oper R = inferior frontal gyrus pars operularis right, OP L = occipital pole left, OP R = occipital pole right, Cereb1R = cerebellum crus 1 right, CerebVI_L = cerebellum 6 left, FP_L = frontal pole left, SFG_L = superior frontal gyrus left, PoNucl_L = left pontine nucleus.

### 3.3 Correlations between ROI-to-ROI connectivity and clinical scores

**Figure 4:**
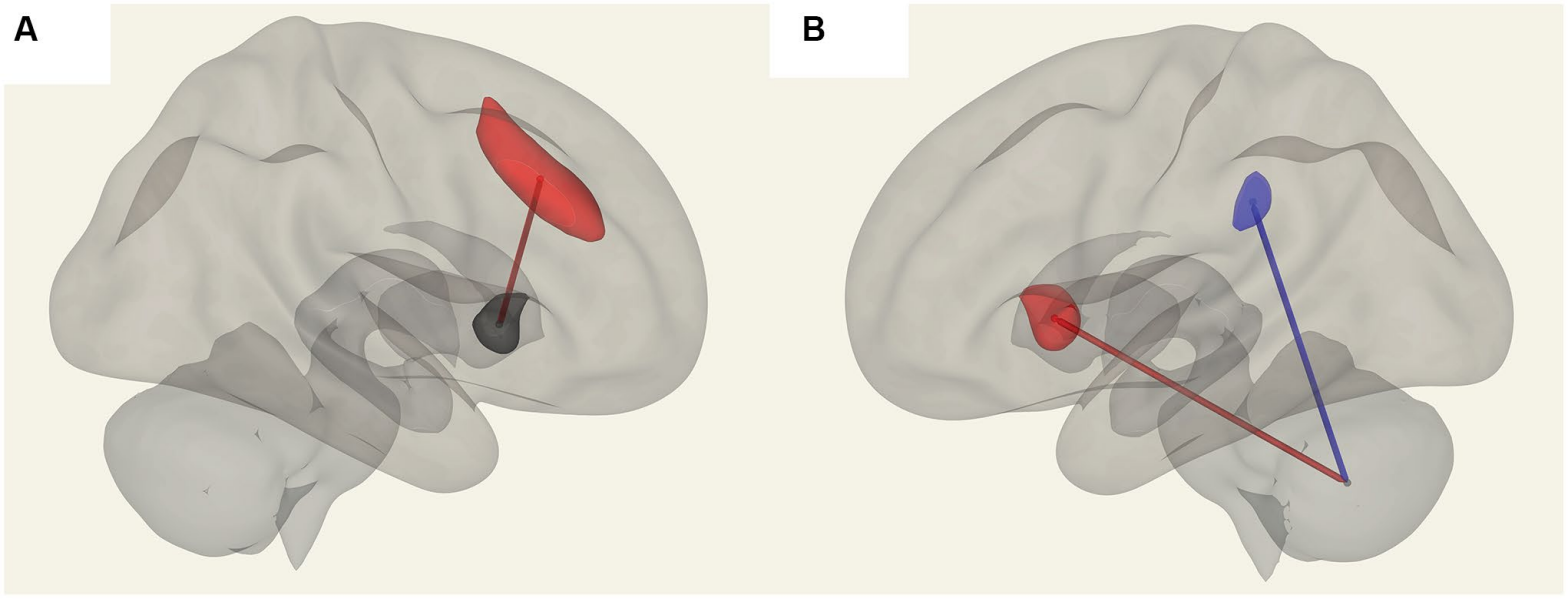
Positive FC (red) correlation with ‘duration of illness’ with the salience network. **(A)** Anterior cingulate gyrus (SA.ACC) to the right insula (SA.Insula). **(B)** Negative correlation with Vermis VIIIb and left supramarginal gyrus (SA.SMG) FC (blue) and a positive correlation with Vermis VIIIb and left insula (SA.Insula) (red) FC.

**Figure 5:**
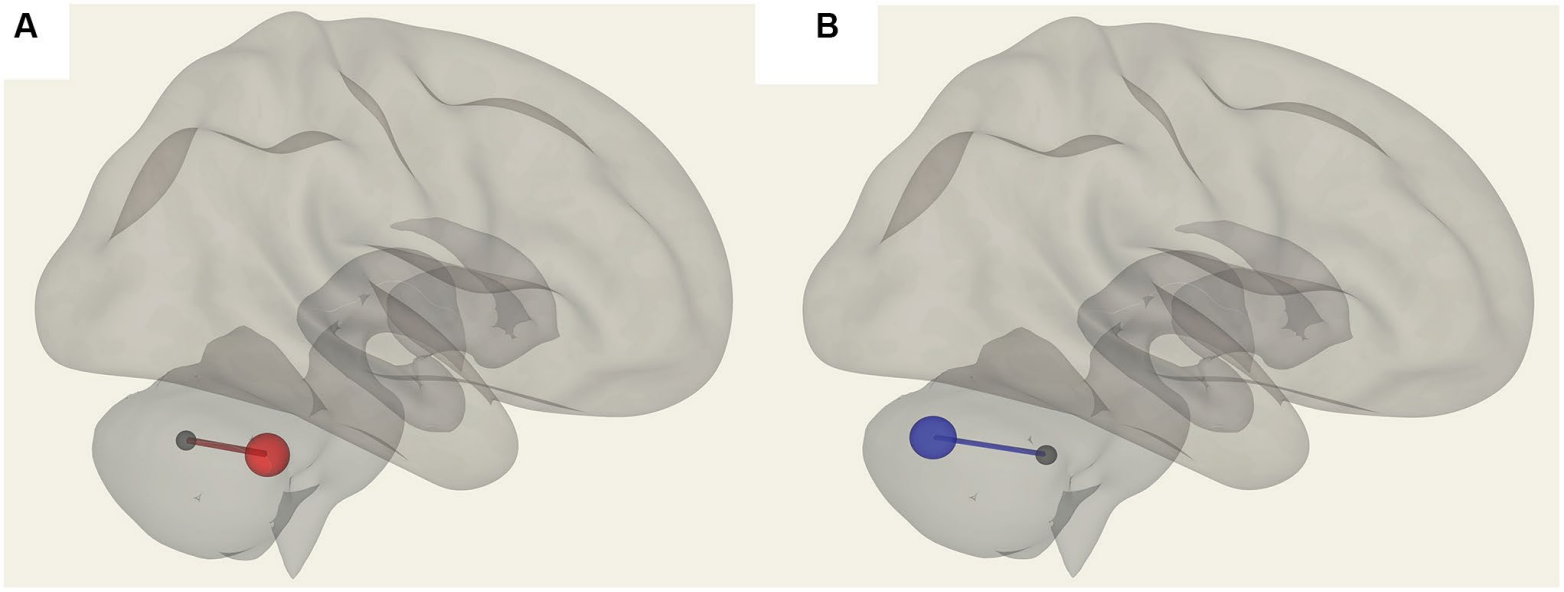
Connections in ME/CFS patients that correlated with memory scores. **(A)** Shows vermis X and vermis VIIb FC, which are positively correlated (in red) and **(B)** shows negative FC correlation with vermis X to vermis crus II (blue).

**Figure 6:**
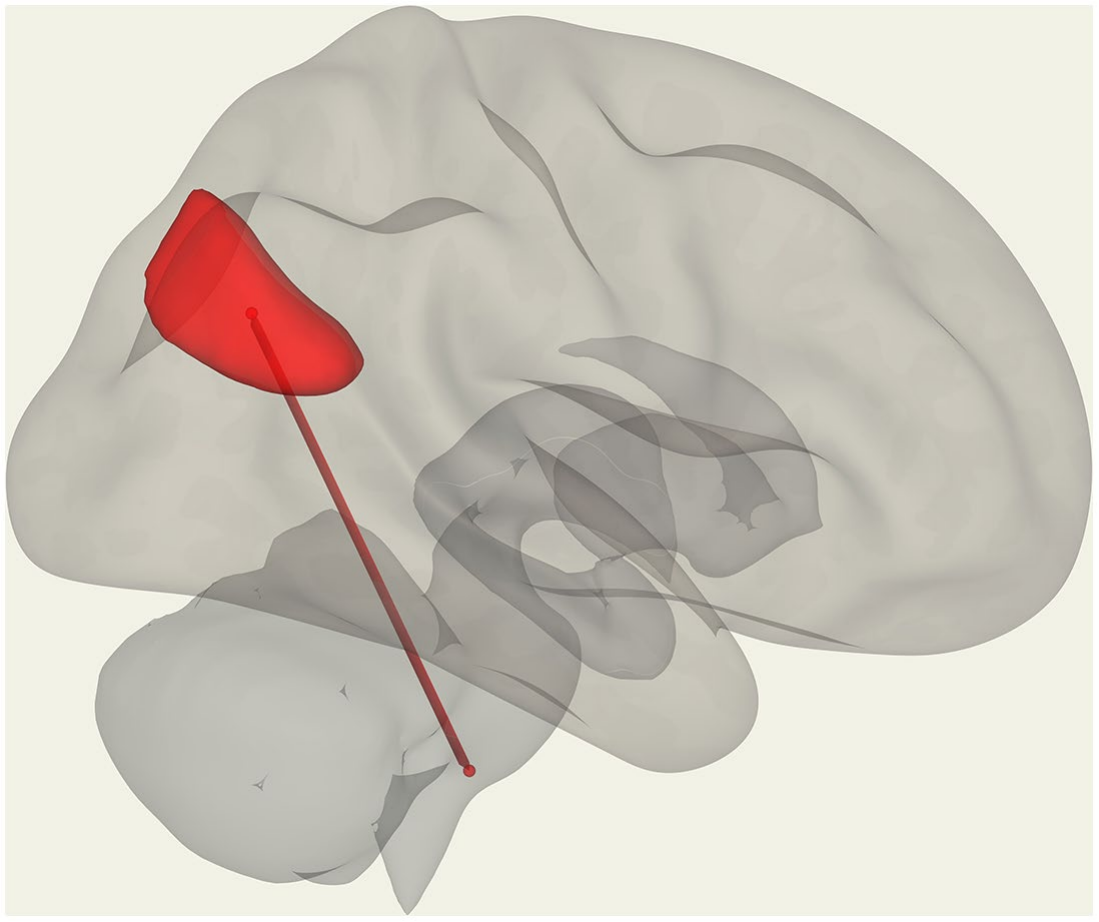
Positive correlation with the respiratory rate (red) in ME/CFS patients for FC between the left medulla and DMN right lateral parietal ROI.

In ME/CFS patients, we found a significant correlation between ROI-to-ROI connectivity and “duration of illness”, “impaired memory”, and “respiratory rate” (Table 4; Figures 4–6). For “duration of illness”, positive FC correlations were observed between the SA anterior cingulate cortex and the right insula (*p* = 0.041) hubs and between the cerebellar vermis VIIIb and left insula (*p* = 0.03), while a negative FC correlation was observed between the same cerebellar vermis VIIIb region and left supramarginal gyrus (SMG) (*p* = 0.030, see Figure 4). For “impaired memory”, a negative correlation was detected with FC between vermis X and vermis crus II (*p* = 0.013), while a positive correlation was detected with FC between vermis X and vermis VIIb (*p* = 0.008, see Figure 5). For “respiratory rate”, a positive correlation was observed with FC between the left medulla and DMN right lateral parietal ROI (*p* = 0.03, see Figure 6).

**Table 4:** ME/CFS connectivity for ROI pairs correlated with “duration of illness”, “impaired memory”, and “respiratory rate”.

ROI 1	ROI 2	p-unc	p-FDR	*R* ^2^
Duration of Illness				
SA.ACC	SA.Insula (R)	0.0015	0.042	0.02
Vermis_VIIIb	SA.SMG (L)	0.0017	0.031	0.304
	SA.Insula (L)	0.0022	0.031	0.270
Impaired memory				
Vermis_VIIb	Vermis_X	0.0030	0.0083	0.267
Vermis_X	Vermis crus_II	0.0005	0.013	0.230
Respiratory Rate				
DMN.LP_R	Mdul_L	0.0014	0.039	0.400

*R*^2^ = adjusted *R* squared. SA.ACC = ant cingulate cortex of salience network, SA.Insula (*R*) = right insula, SA.SMG (L) = left supramarginal gyrus, SA.Insula (L) = left insula, DMN.LP (*R*) = right Lateral parietal of the default mode network, Mdul_L = left medulla.

## 4 Discussion

In this study, we investigated functional connectivity differences in brain regions for ME/CFS patients and HCs using 7T MRI. We also explored FC differences for ROIs and clinical scores available for the duration of illness, impaired memory, and respiratory rates. This is the first study to report FC differences in ME/CFS patients using ultra high-field MRI.

### 4.1 ROI-to-ROI analysis

We compared 31 ME/CFS patients and 15 HC individuals and found reduced FC in ME/CFS patients between vermis IX of the cerebellum and the PoNucl in the brainstem. The cerebellum is involved in the maintenance of balance and the coordination of voluntary movements, eye movements, motor learning, and cognitive functions (Nagao, 2004). The cerebellar vermis lies in the medial cortico-nuclear zone of the cerebellum, and lesions of the vermis have been reported to cause deficits in whole-body posture and locomotion (Chambers and Sprague, 1955). The cerebral cortex and the cerebellum communicate through the pontine nuclei in the pons (PoNucl), which serves as a hub circuit between the cerebrum, pons, and cerebellar peduncle (Middleton and Strick, 2000; Leergaard et al., 2004). The pontine nucleus supports the execution of skilled movement by linking the motor cortex and the cerebellum (Nagao, 2004). Hence, disruption in pontine nuclei functions would impair movement execution.

We also observed stronger connectivity between the pontine nuclei and clusters of cortical voxels in the frontal pole (FP, left) and superior frontal gyrus (SFG, left). Both frontal regions are involved in self-awareness, integrating sensory system information, and initiating and monitoring the appropriate response (Koechlin et al., 2002; Daw et al., 2006), and in our case, a motor response to the Stroop task. In ME/CFS patients, stronger FC between the pontine nuclei and frontal regions during the Stroop task could be due to diminished frontal function demanding hyper-stimulation to complete the task.

### 4.2 Seed-to-voxel analysis

Our seed-based analysis detected reduced connectivity in ME/CFS between the left cuneiform nucleus ROI and occipital poles and cerebellum I regions and between the culmen ROI and cerebellum VI (see Table 3). The cuneiform nucleus is a midbrain structure and part of the reticular activation system (RAS). The RAS nuclei of the brainstem are fundamental for regulating the sleep/wakefulness cycles and consciousness (Mesulam, 1995; Garcia-Rill et al., 2013) and are believed to influence locomotor function and autonomic responses. The cuneiform nucleus, along with other RAS nuclei, has been implicated in determining signals for blood pressure and other autonomic effectors and has been reported to be altered in ME/CFS (Barnden et al., 2016). The left cuneiform was also highlighted in an intra-brainstem connectivity study indicating FC deficits in the brainstem region (Barnden et al., 2019). Another study indicated variations in myelin and/or iron (T1wSE) in the cuneiform nucleus. These RAS regions together with the cerebellum can influence abnormal blood pressure, autonomic function, sleep quality, and cortical arousal levels, affecting memory, learning, and problem-solving, which appear to be impaired in ME/CFS patients (Barnden et al., 2019). The role of occipital regions, however, requires further investigation in ME/CFS patients. We also observed stronger FC within the DMN in ME/CFS patients. The DMN connects the cuneus-precuneus, posterior cingulate cortex (PCC), medial frontal cortex, and inferior parietal regions (Gusnard and Raichle, 2001; Fox and Raichle, 2007; Greicius et al., 2009) to constitute one of the primary intrinsic networks of the brain essentially responsible for the brain’s baseline functions. The DMN mediates the processing of one’s thoughts and feelings (Raichle and Snyder, 2007) and mind wandering, with its FC correlating with overall cognitive performance (Persson et al., 2014; Mak et al., 2017). The cuneus-precuneus cortex has been identified as being functionally variable, showing varying connectivity with different networks according to the task state, suggesting its important role in integrating external and internal information (Utevsky et al., 2014). FC networks involving cortical and subcortical regions along with the anterior and posterior cingulate and the cerebellum are in agreement with literature that the cuneus-precuneus cortex is involved in the DMN activity and is functionally connected (Mak et al., 2017). Increased FC has been proposed to relate to a compensation response to the early degeneration process, especially involving cognitive function (Byun et al., 2020). Consequently, in ME/CFS patients, a strong FC between the PCC and cuneus-precuneus could be due to upregulated connectivity to compensate for the diminished function of the cortico-pontine pathway, thereby resulting in increased processing of input and hyper-alertness during the cognitive task in ME/CFS patients (Boissoneault et al., 2016).

### 4.3 FC correlations with clinical measures

The association of ROI-to-ROI FC with the clinical score “duration of illness” in ME/CFS patients demonstrated a positive correlation within and from salience network (SA) hubs (ACC, bilateral anterior insula, and left supramarginal gyrus). Positive correlations were observed with FC between the salience network’s anterior cingulate cortex (ACC) and right insula, left insula, and vermis VIIIb, and left supramarginal gyrus (SMG) and vermis VIIIb (Table 4). The salience network comprises the insular cortex and the ACC and is involved in interoception, the detection of salient stimuli, pain, and deception (Borsook et al., 2013; Kucyi and Davis, 2015). It controls the relative activity of the DMN and central executive network (CEN), providing a transitional link for cognition and emotional awareness (Lois et al., 2014). As part of the limbic system, the ACC and insula have been associated with fatigue (Kohl et al., 2009; Staud et al., 2018). The ACC and the superior medial frontal regions are key in providing energy in attention-demanding tasks (Nordin et al., 2016), and the ACC has been reported to play a vital role in performance monitoring and cognitive control (Lorist et al., 2005). Therefore, dysfunction in the ACC and frontal areas in ME/CFS patients is consistent with sluggish performance in cognitive tasks, particularly those demanding sustained attention and fast responses. Hypoperfusion has been reported in the ACC that could result in impaired cognitive control of the brain in ME/CFS patients, implying an impaired ability to evaluate or adapt to the energy demands of task performance, resulting in fatigue (Li et al., 2021). Insular dysfunction and its association with fatigue severity and pain intensity in ME/CFS patients (Wortinger et al., 2016) suggests that an anomaly in the salience network may be central to the pathomechanism.

The self-reported “impaired memory score” was positively correlated with the FC among cerebellar regions in ME/CFS patients, which is consistent with cerebellar involvement in memory dysfunction (Marvel and Desmond, 2010). A recent PET study has reported underlying severe and extensive hypometabolism in the cerebellum region in patients with long-lasting ME/CFS (Sahbai et al., 2019). A recent study reported that memory deficits in ME/CFS patients were associated with hippocampal volume changes (Thapaliya et al., 2022). Another study in ME/CFS patients detected diffusion deficits in the fronto-pontine tract (Thapaliya et al., 2021), supporting a role for the pontine nucleus and cerebellum in motion execution, relating it to impaired motor response preparation for some tasks (Paul et al., 2008; Rasouli et al., 2017).

The “respiratory rate” clinical score was positively correlated with FC between the medulla of the brainstem and the DMN’s inferior lateral parietal (LP) hub. Activation of these two regions is consistent with the findings (Barnden et al., 2023) in a study of long COVID patients where they established increased medullary–DMN connectivity, which will facilitate executive network activity (Barnden et al., 2023). A DTI study showed microstructural correlations with the respiratory rate in the cerebellar tonsil and superior prefrontal cortex in ME/CFS patients (Thapaliya et al., 2021). Respiration and other autonomic measures are regulated by the central autonomic network, which involves several cortical regions along with the amygdala, hypothalamus, midbrain, pons, and medulla (Benarroch, 1993). Therefore, cerebellar involvement is integral to respiratory function (Parsons et al., 2001; Macey et al., 2005).

## 5 Limitations and prospects

Clinical scores reported in this investigation were self-reported. Given that this was a cross-sectional study, future longitudinal investigation may provide greater insights into the underlying pathomechanism in ME/CFS patients. A prospective study should be conducted with a larger sample size of HCs with age- and sex-matched ME/CFS subjects.

## 6 Conclusion

Our findings showed impaired FC in ME/CFS patients. We found complex connections, both stronger and reduced in ME/CFS patients compared to HCs. Furthermore, we also demonstrated that symptom severity scores of ME/CFS were associated with FC in diverse brain regions. Therefore, the widespread involvement of cortical regions and the ponto-cerebellar circuit is consistent with the symptomatology of ME/CFS. The results highlight and suggest possible ongoing changes in ME/CFS individuals, particularly in the pons and cerebellum areas, providing insights into the pathophysiology of altered cognition and disability in ME/CFS.

## Data availability statement

The original contributions presented in this study are included in this article/Supplementary material, further inquiries can be directed to the corresponding author.

## Ethics statement

The studies involving humans were approved by Griffith University Human Research Ethics Committee (2022/666). The studies were conducted in accordance with the local legislation and institutional requirements. The participants provided their written informed consent to participate in this study.

## Author contributions

MI: Conceptualization, Formal analysis, Investigation, Methodology, Software, Writing – original draft, Writing – review & editing. KT: Supervision, Writing – review & editing, Data curation. SM-G: Funding acquisition, Project administration, Resources, Supervision, Validation, Writing – review & editing. MB: Methodology, Writing – review & editing. LB: Methodology, Supervision, Validation, Writing – review & editing.

## Figures and Tables

**Table:** 

*Precuneous*	precuneous cortex
*IFG oper R*	inferior frontal gyrus pars operularis right
*Cereb2 L*	cerebellum crus2 left
*Cuneal R*	cuneal cortex right
*OP L*	occipital pole left
*OP R*	occipital pole right
*Cereb1R*	cerebellum crus 1 right
*iLOC_L*	lateral occipital cortex inferior division left
*Cuneal R*	cuneal cortex right
*ICC R*	intracalcarine cortex right
*Ver7*	vermis 7
*FP_L*	frontal pole left
*SFG_L*	superior frontal gyrus left
*IFG tri L*	inferior frontal gyrus pars triangularis Left
*Med FC*	frontal medial cortex
*Caudate L*	caudate left
*Cereb9 R*	cerebellum 9 right
*PaCiG L*	paracingulate gyrus left
*MidFG R*	middle frontal gyrus right
*IFG_oper_L*	inferior frontal gyrus pars opercularis left
*aSTG_L*	superior temporal gyrus anterior division left
*PaCiG_L*	paracingulate gyrus left
*Cereb6_L*	cerebellum 6 left.
