# Supplementary material for: Subcortical and default mode network connectivity is impaired in myalgic encephalomyelitis/chronic fatigue syndrome
Source: Front Neurosci. 2024 Jan 29;17:1318094. doi: 10.3389/fnins.2023.1318094 (PMC10859529; doi:10.3389/fnins.2023.1318094)
Supplement: Supplementary file 1 [file Data_Sheet_1.PDF]

Linear Graphs for correlation analyses for Duration of illness, impaired memory scores and respiratory rates in 31 ME/CFS

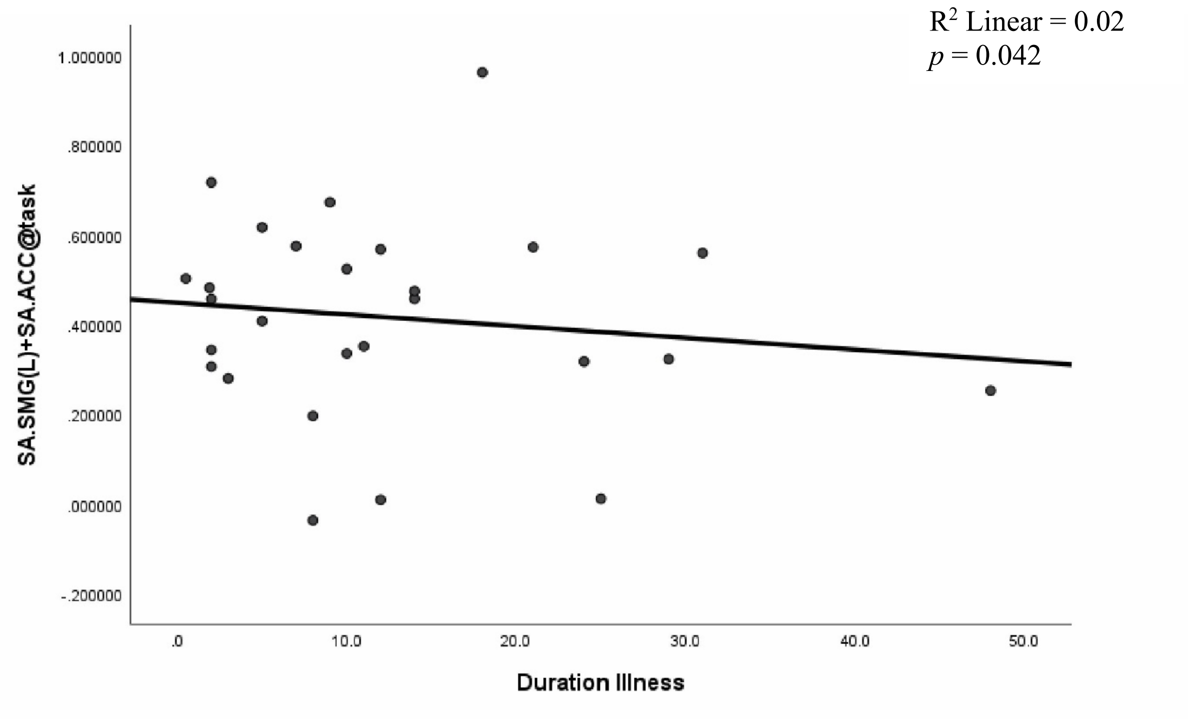

Figure 1. shows the correlation between Duration of illness (x-axis) and functional connectivity between SA.SMG and SA.ACC (y-axis)

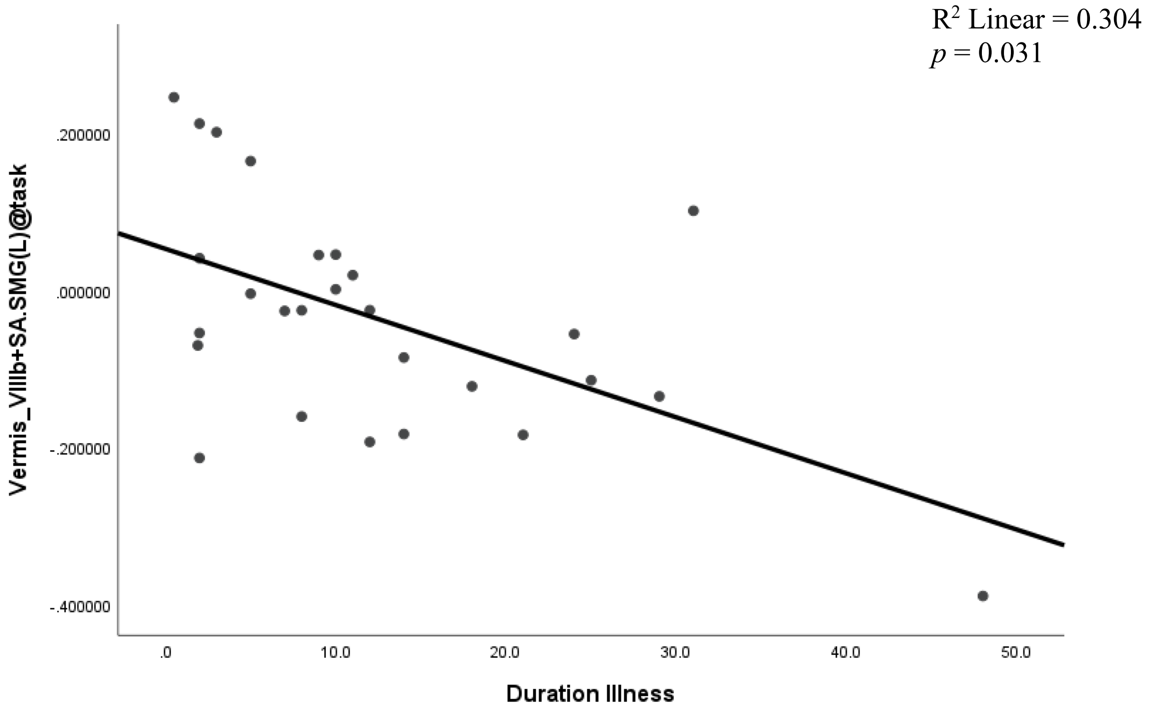

Figure 2. shows the correlation between Duration of illness (x-axis) and functional connectivity between vermis_VIIIb and SA.SMG (y-axis)

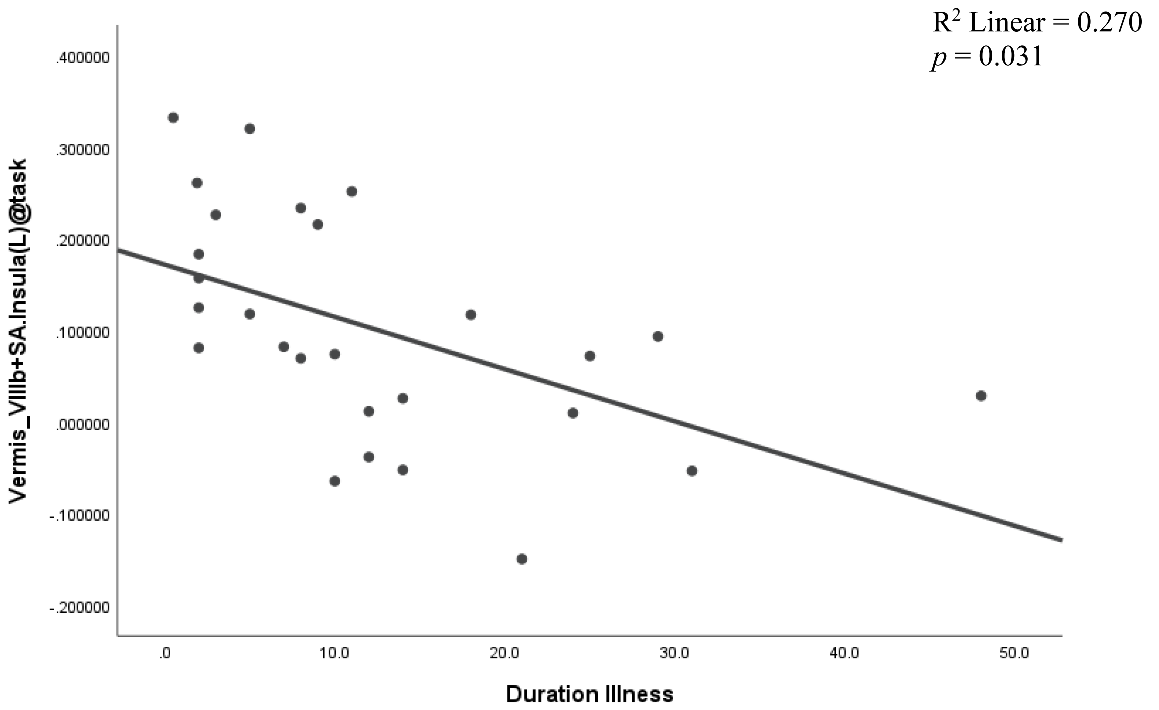

Figure 3. shows the correlation between Duration of illness (x-axis) and functional connectivity between Vermis_VIIIb and SA.Insula_left (y-axis)

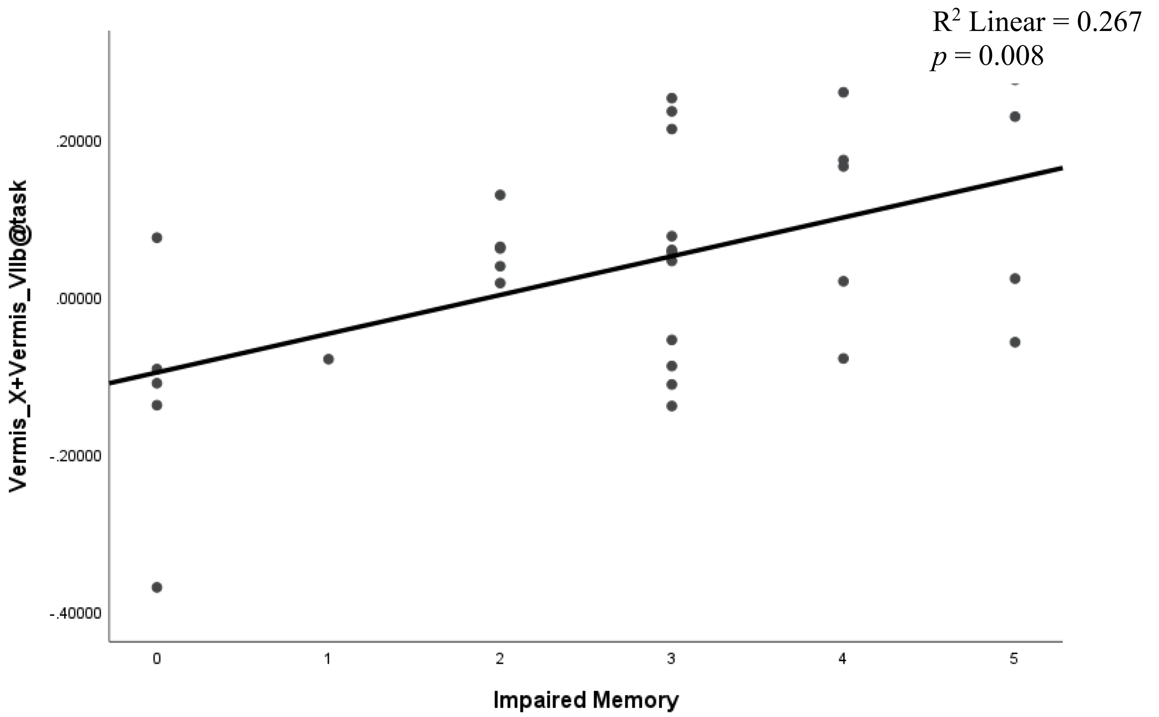

Figure 4. shows the correlation between Impaired Memory (x-axis) and functional connectivity between Vermis_X and Vermis_VIIb (y-axis)

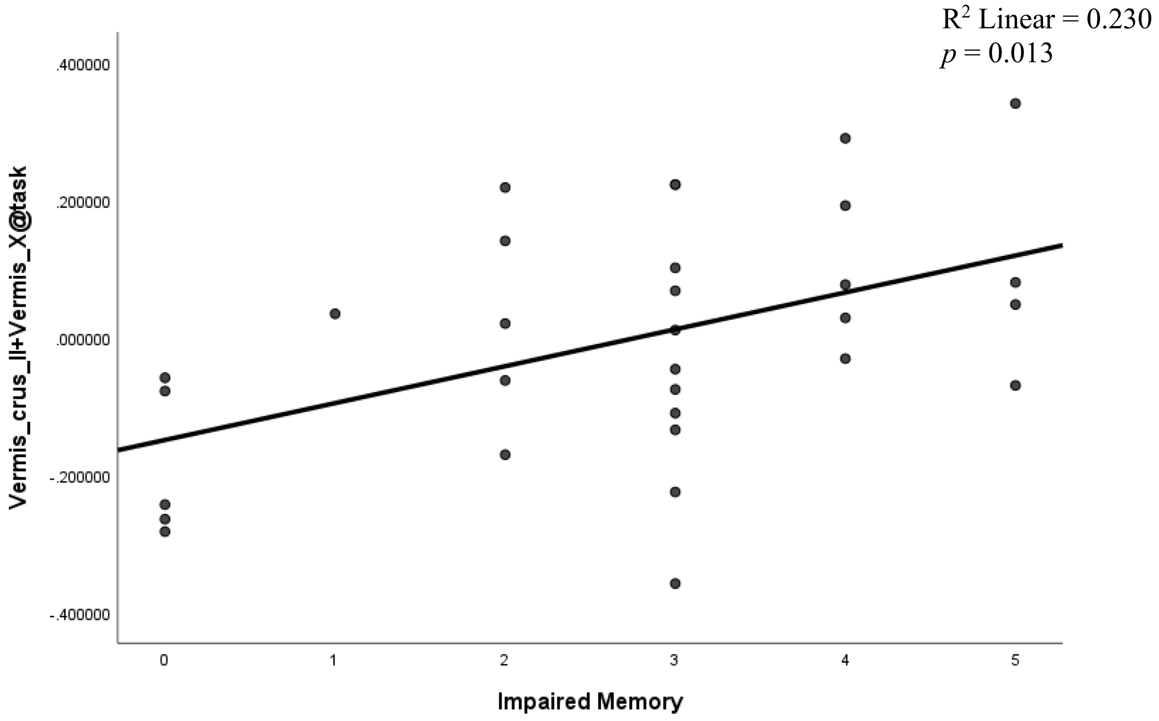

Figure 5. shows the correlation between Impaired Memory (x-axis) and functional connectivity between Vermis_crusII and Vermis_X (y-axis)

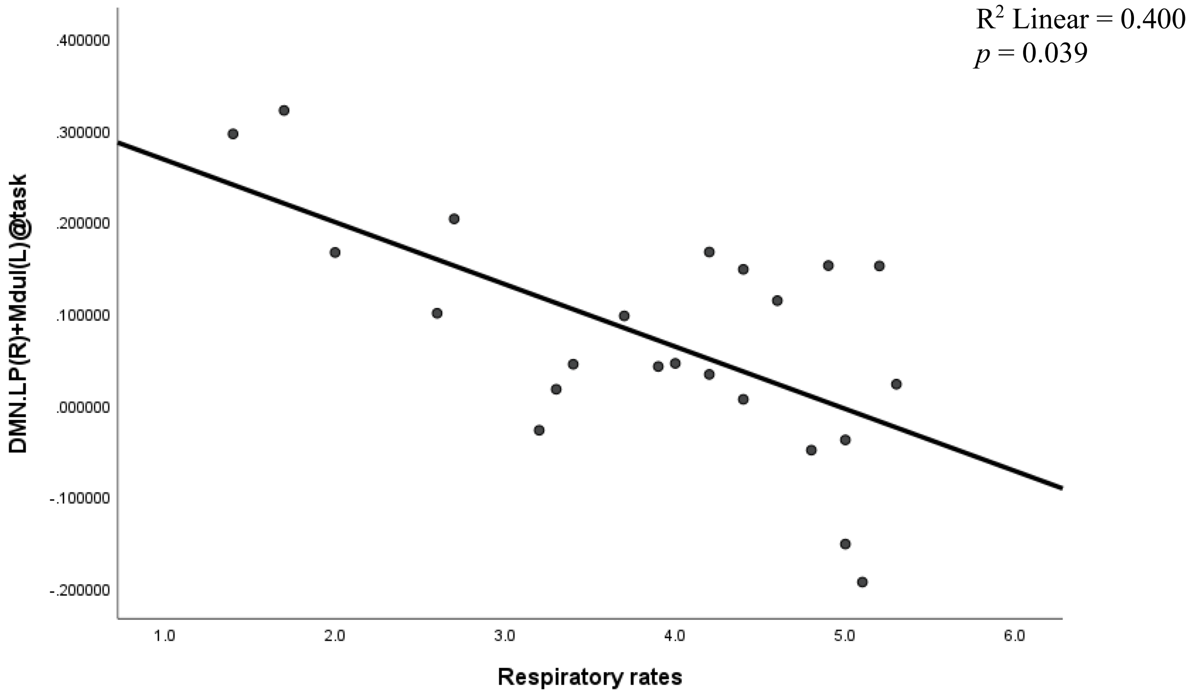

Figure 6. shows the correlation between Respiratory rates (x-axis) and functional connectivity between DMN.LP_right and Medulla_left (y-axis)

Table 1. lists subjects with age, gender for both HC & ME/CFS, and illness length (in years), impaired memory scores and respiratory rates for ME/CFS patients. N/A denotes not available.

Sub ID	Age	Gender	Illness Duration	Impaired memory	Respiratory rates
1	38.1	1	0	0	0
2	33.2	2	0	0	0
3	21.7	1	0	0	0
4	36.5	2	0	0	0
5	58.7	1	0	0	0
6	25.4	2	0	0	0
7	43.3	1	0	0	0
8	22.2	2	0	0	0
9	49.2	1	0	0	0
10	50.1	1	0	0	0
11	22.7	2	0	0	0
12	47.1	1	0	0	0
13	59.9	1	0	0	0
14	32.4	1	0	0	0
15	33.5	1	0	0	0
16	23.5	1	7	2	4.6
17	25.6	1	2	2	NaN
18	61.4	2	18	0	2.7
19	36.6	1	21	5	3.7
20	45.3	1	10	0	NaN
21	52.3	1	5	3	1.4
22	38.5	1	12	0	4.9
23	47.6	1	9	3	3.9
24	46.8	1	2	0	2
25	30.3	2	2	3	5
26	60.1	1	11	5	3.2
27	39	1	25	5	4
28	52.1	1	10	3	2.6
29	53.1	1	5	3	NaN
30	30.6	1	14	4	1.7
31	44.1	1	8	3	4.2
32	48.4	1	29	3	4.2
33	45.6	1	24	2	5.2
34	55.6	1	48	5	5.1
35	24.1	1	3	2	4.4
36	59.8	1	12	4	4.8
37	32	2	2	2	NaN
38	46.5	2	8	0	5.3
39	31	1	14	4	5
40	49	1	0.5	3	3.3
41	30	1	1.9	3	3.4
42	55	1	31	4	4.4
43	51.6	2	NaN	3	NaN

44	43.6	2	NaN	1	NaN
45	46.9	2	NaN	4	NaN
46	32.3	1	NaN	3	NaN
